# Cost-effectiveness of immediate versus delayed sequential bilateral cataract surgery in the Netherlands (the BICAT-NL study): study design of a prospective multicenter randomised controlled trial

**DOI:** 10.1186/s12886-020-01521-x

**Published:** 2020-06-29

**Authors:** L. S. Spekreijse, R. W. P. Simons, B. Winkens, F. J. H. M. van den Biggelaar, C. D. Dirksen, R. M. M. A. Nuijts

**Affiliations:** 1 Maastricht University Medical Center+, University Eye Clinic Maastricht, P. Debyelaan 25, 6229 HX Maastricht, the Netherlands; 2 School for Mental Health and Neuroscience (MHeNs), Maastricht University, Maastricht, the Netherlands; 3 Department of Methodology and Statistics, Faculty of Health, Medicine and Life Sciences (FHML), Care and Public Health Research Institute (CAPHRI), Maastricht University, Maastricht, the Netherlands; 4 Department of Clinical Epidemiology and Medical Technology Assessment, CAPHRI School for Public Health and Primary Care, Maastricht University Medical Center+, Maastricht, the Netherlands; 5 Department of Ophthalmology, Zuyderland Medical Center, Heerlen, the Netherlands

**Keywords:** Immediately sequential bilateral cataract surgery (ISBCS), Delayed sequential bilateral cataract surgery (DSBCS), Randomized controlled trial, Cost-effectiveness, Refraction, Complications, Visual acuity, Patient reported outcome measures (PROMs)

## Abstract

**Background:**

Cataract surgery is one of the most frequently performed types of surgery. Most patients suffer from bilateral cataract and while cataract surgery of only one eye is effective in restoring functional vision, second-eye surgery leads to further improvements in health-related quality of life, and is cost-effective. At present, most patients undergo cataract surgery in both eyes on separate days as recommended in national guidelines, referred to as delayed sequential bilateral cataract surgery (DSBCS). An alternative procedure involves operating both eyes on the same day, but as separate procedures, known as immediately sequential bilateral cataract surgery (ISBCS). The aim of this study is to evaluate the effectiveness and costs of ISBCS compared to DSBCS, in order to test the hypothesis that ISBCS is non-inferior to DSBCS in terms of effectiveness and superior to ISBCS in terms of cost-effectiveness.

**Methods/design:**

Multicenter non-inferiority randomised controlled clinical trial. Patients (18 years or older) with bilateral cataract and an indication for bilateral cataract surgery with an expected uncomplicated intraoperative and postoperative course are included in the study. Patients are randomly assigned to either ISBCS or DSBCS. The primary endpoint is the proportion of patients with a refractive outcome in the second eye within 1.0 dioptre from the target refraction, at 4 weeks after surgery. Secondary outcomes include corrected and uncorrected distance visual acuity, complications, patient reported outcomes (PROMs), cost-effectiveness, and budget impact. Follow-up visits are planned at 1 week after first-eye surgery and 4 weeks after second-eye surgery. At 3 months after first-eye surgery, the occurrence of complications is checked and patients fill in a final questionnaire.

**Discussion:**

This study protocol describes the design of a multicenter non-inferiority randomised controlled trial. Current studies on ISBCS often lack information on safety regarding refractive outcomes. In addition, there is a lack of well-designed cost-effectiveness studies using established methods. The BICAT-NL study will provide more insight in refractive and cost-effectiveness outcomes for ISBCS compared to DSBCS.

**Trial registration:**

This study was prospectively registered at Clinicaltrials.gov on January 17th 2018. (Identifier: NCT03400124.

## Background

Cataract surgery is one of the most frequently performed types of surgery in the world. In the Netherlands, the current estimated number of cataract extractions is over 180,000 per year [1, 2]. This number has increased rapidly over the years. As a result, national healthcare expenditures on cataract have increased as well. Due to the vast number of cataract patients, small efficiency gains in cataract care delivery may lead to substantial cost savings on a macro level.

The majority of patients, mostly elderly, suffer from bilateral cataract. A previous study showed that 71% of patients with cataract had an indication for bilateral surgery [3]. While cataract surgery of only one eye is effective in restoring functional vision, studies have shown that cataract surgery of the second eye leads to further improvements in health-related quality of life, and is cost-effective [4–6].

At present, most patients undergo cataract surgery in both eyes on separate days, as advised in the Cataract guidelines of the Dutch Ophthalmology Society, with a delay of at least 2 weeks (delayed sequential bilateral cataract surgery, DSBCS) [7]. However, over the past years some have argued that the procedure is now safe enough to perform in both eyes on the same day during a single operating session (immediately sequential bilateral cataract surgery, ISBCS) [8]. These advocates substantiate their attitudes to ISBCS through the many advances in the field of cataract surgery and consider ISBCS to be safe when risks of complications related to surgical procedures are minimized [9].

The two primary reasons for delaying second-eye surgery are the risk of bilateral endophthalmitis and the risk of refractive surprise. Endophthalmitis is most likely to occur within the first 2 weeks after surgery. When this happens, cataract surgeons may decide to abstain from second-eye surgery. However, unilateral endophthalmitis is a rare complication, especially since the introduction of intracameral antibiotic prophylaxis. Several retrospective and epidemiologic studies have reported that the administration of intracameral antibiotics significantly reduces the risk for developing endophthalmitis compared to other prophylactic measures, such as topical antibiotics [10, 11]. More recent studies on incidences of endophthalmitis after administration of intracameral antibiotics, show endophthalmitis rates of 0.039% (Spain) [12] and 0.029% (Sweden) [13]. In addition, the study on Swedish national data reports a significant decrease in endophthalmitis rates compared to previous years [13]. The calculated probability of endophthalmitis occurring bilaterally is extremely low (1: 70 million cases) [8]. So far, the few cases of bilateral endophthalmitis after ISBCS that have been described could be attributed to faults in aseptic procedures [14]. Three randomised controlled trials (RCTs) [15–17] and several non-comparative studies [18–26] found no significant differences in (severe) complication rates. With regard to refractive outcomes, delaying second-eye surgery enables cataract surgeons to evaluate the outcomes of the first eye and, if necessary, adjust their plans for second-eye surgery [27]. In the Netherlands, the standard for success that is used in cataract surgery is a postoperative refraction within 1.0 diopter of the target refraction [28]. Only two previous studies, one randomised study and one nonrandomised comparative study, [15, 29] report data on refractive outcomes for ISBCS compared to DSBCS. These studies showed similar refractive outcomes for ISBCS compared to DSBCS, indicating that refractive surprises may be prevented with careful patient selection [15, 25, 29, 30]. However, the number of patients in these two studies was limited and the overall quality of the evidence of the RCT was graded low to moderate [31]. Therefore, concerns remain with regard to refractive outcomes.

Furthermore, available studies on cost analyses showed that ISBCS resulted in fewer costs and important cost savings to third-party payers, patients, and society compared to DSBCS [3, 32–36]. However, to date, only one (model-based) cost-utility analysis has been performed [36].

Therefore, the aim of the BICAT-NL (Bilateral Cataract surgery in the Netherlands) study is to evaluate the effectiveness and costs of ISBCS compared to DSBCS in the Netherlands in order to test the hypotheses that ISBCS is non-inferior to DSBCS in terms of effectiveness and superior to ISBCS in terms of cost-effectiveness.

## Methods/design

### Objectives

The primary objective of this study is to evaluate whether ISBCS is non-inferior to DSBCS regarding effectiveness, where effectiveness is defined as the proportion of patients with a postoperative refraction that deviates ≤1 D from target refraction. Secondary objectives of this study are to evaluate ISBCS versus DSBCS regarding non-inferiority of (1) the proportion of patients with a postoperative refraction within 0.5 D of target refraction, and (2) postoperative visual acuity, and superiority of (3) patient satisfaction (using patient reported outcome measures (PROMs)), (4) the incidence of complications, and (5) cost-effectiveness.

### Study design and setting

A multicenter non-inferiority randomized controlled clinical trial will be performed at outpatient ophthalmology clinics in one academic center (Maastricht University Medical Center (MUMC+)) and nine non-academic centers in the Netherlands (Zuyderland Medical Center, Heerlen; Canisius Wilhelmina Hospital, Nijmegen; Gelre Hospital, Zutphen; Deventer Hospital, Deventer; Elisabeth TweeSteden Hospital, Tilburg; Amphia Hospital, Breda; Medical Center Haaglanden, Den Haag; Medical Spectrum Twente, Enschede; Isala Clinic, Zwolle).

### Study population

**Fig. 1:**
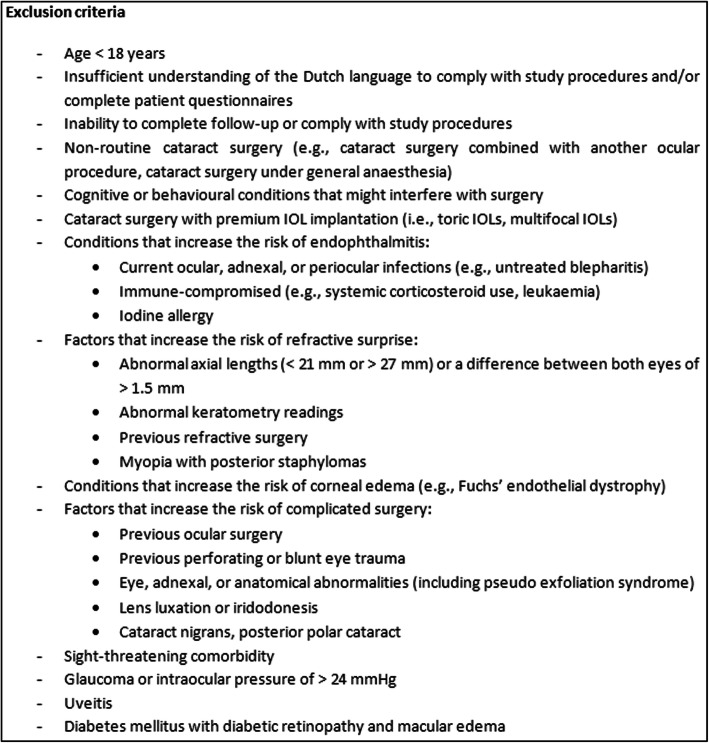
Exclusion criteria

All patients with bilateral cataracts undergoing expected uncomplicated bilateral cataract surgery using a standard phacoemulsification technique will qualify for inclusion in this study. The exclusion criteria are listed in Fig. 1.

### Study procedures

Visits are planned at baseline (preoperatively), and 1 week after first-eye surgery and 4 weeks after second-eye surgery. Patients will undergo study procedures as shown in Table 1. At 3 months after surgery, the occurrence of complications is derived from the patient record and patients will fill in the final questionnaires. Visual acuity will be measured by using the Early Treatment Diabetic Retinopathy Study (ETDRS) chart and measured refraction will be subjective (not automated).

**Table 1:** Study procedures per follow-up time point

Assessment/ procedure	Visit					
	Preoperative	Surgery 1	1 week after surgery 1	Surgery 2 (if applicable)	4 weeks after surgery 2	3 months after surgery 1 (post/email)
Check for in−/exclusion criteria	X					
Informed Consent	X					
Medical history	X					
Check medication	X	X	X	X	X	X^a^
Ophthalmic examination	X		X		X	
Check adverse events		X	X	X	X	X^a^
Refraction	X		X		X	
CDVA	X		X		X	
NEI VFQ-25 questionnaire	X					X
Catquest-9SF questionnaire	X					X
EQ-5D-5L questionnaire	X		X		X	X
HUI-3 questionnaire	X		X		X	X
Cost questionnaire	X					X

^a^derived from the patient record and questionnaires

The intervention consists of bilateral cataract surgery by an experienced cataract surgeon. A standard phacoemulsification technique with intraocular lens implantation will be used. Surgery will be performed either during a single operating session (ISBCS) or during separate operating sessions with a minimum of 2 weeks apart (DSBCS), depending on randomisation. ISBCS will be performed in accordance with the “iSBCS General Principles for Excellence in ISBCS 2009”, treating both eyes as completely separate aseptic procedures in order to minimize the risk of bilateral endophthalmitis. For both ISBCS and DSBCS, intracameral antibiotics will be administered at the end of each operation. Topical antibiotics and anti-inflammatory drugs will be commenced postoperatively. After surgery, DSBCS patients will receive an eye patch and ISBCS patients will receive a transparent eye shield.

Patients can withdraw from the study at any time for any reason without any consequences. The investigator can decide to withdraw a patient from the study for urgent medical reasons. In case a patient withdraws from the study after randomization, the case is considered lost to follow-up, but will be included in the intention to treat analysis.

### Outcome measures

The primary outcome is the proportion of patients in both treatment groups with a postoperative refraction in the second eye that deviates ≤1.0 D from target refraction 4 weeks after cataract surgery of the second eye. This is an accepted norm to define success in terms of refractive outcomes after cataract surgery [7, 28].

Secondary outcomes will include:

1. **Refraction**: the proportion of patients in both treatment groups with a postoperative refraction in the second eye that deviates ≤0.5 D from target refraction 4 weeks after cataract surgery of the second eye.
2. **Visual acuity**: ETDRS, uncorrected and corrected distance visual acuity in LogMAR (the Logarithm of the Minimum Angle of Resolution), 1 week after first-eye surgery and 4 weeks after second-eye surgery, corrected for baseline visual acuity. In addition, the proportion of patients with a final corrected distance visual acuity (CDVA) of ≤0.1 LogMAR (measured at 4 weeks after second-eye surgery) will be compared for ISBCS and DSBCS.
3. **Complications**: the number of intraoperative and postoperative complications within 3 months after first-eye surgery.
4. **Patient-reported outcome measures (PROMs)**, including patient satisfaction, vision-specific quality of life, and health-related quality of life:
  - Vision-specific quality of life and patient satisfaction will be measured using the National Eye Institute Visual Function Questionnaire-25 (NEI VFQ-25) [37] and the Catquest-9SF [38], measured at baseline and 3 months after first-eye surgery (Table 1).
  - Health-related quality of life (HRQL) will be measured using two questionnaires: EuroQol’s EQ-5D-5L [39] and the Health Utilities Index Mark-3 (HUI-3) [40], measured at baseline, 1 week after first-eye surgery, 4 weeks after second-eye surgery and 3 months after first-eye surgery (Table 1).
5. **Incremental cost-effectiveness ratios (ICERs)**: these will be expressed as 1) incremental societal costs per quality-adjusted life year (QALY) gained, 2) incremental healthcare costs per patient with postoperative refraction ≤1.0 D from target refraction, 3) incremental healthcare costs per clinically improved patient on the NEI VFQ-25 questionnaire, 4) incremental healthcare costs per clinically improved patient on the Catquest-9SF questionnaire, and 5) incremental healthcare costs per patient with clinical improvement in (un)corrected distance visual acuity. QALYs will be calculated based on generic HRQL.
6. **Budget impact** will be reported as a difference in costs. Different scenarios will be compared to investigate the impact of various levels of implementation.

Adverse events are defined as any undesirable ophthalmic event occurring during the study, whether or not considered related to the trial procedure or the experimental intervention. All ophthalmic adverse events reported spontaneously by the patient or observed by the investigator or his staff will be recorded. Serious adverse events are defined as any untoward medical occurrence or effect that results in death, is life threatening (at the time of the event), requires hospitalisation or prolongation of existing inpatients’ hospitalisation, results in persistent or significant disability or incapacity, is a congenital anomaly or birth defect, or any other important medical event that did not result (but could have resulted) in any of the outcomes listed above due to medical or surgical intervention.

### Randomisation and blinding

Study participants will be randomized to either ISBCS (intervention) or DSBCS (control). Each patient will receive a randomization number from a computerized random number generator. In a certified electronic data capture tool called Castor EDC [41], the subject number will be allocated to ISBCS or DSBCS through block randomisation stratified for center and axial length. Random varying block sizes of 2 and 4 will be used. Blinding is not possible because of the nature of the intervention.

### Sample size calculation

The sample size calculation is based on the proportion of patients with a postoperative refractive error in the second eye that deviates less than 1.0 D from target refraction. The only RCT thus far that reported this outcome showed that 91.0 and 90.3% of ISBCS and DSBCS eyes, respectively, was within 1.0 D [15]. Another (nonrandomized) comparative study showed that 96.8 and 97.0% of ISBCS and DSBCS eyes, respectively, was within 1.0 D [29]. Since no difference is expected with regard to this outcome, these percentages were averaged giving an expected proportion of 94% in both treatment groups. For the sample size calculation, it is assumed that the proportion in the ISBCS group is equal to or smaller than the proportion in the DSBCS group (i.e., non-inferiority). A non-inferiority margin of 5% is allowed (i.e., the proportion in ISBCS would need to be > 5% lower than the proportion in DSBCS to detect a statistically significant difference). The probability of a Type I error (alpha) is set at 0.05, and the probability of a Type II error (beta) is set at 0.10 (i.e., power is 90%). All sample sizes were calculated using an online 2-sample non-inferiority or superiority calculator for the comparison of two proportions (www.powerandsamplesize.com). The required sample size is 386 patients per group or 772 patients in total. Factoring in a loss to follow-up of 10% gives a final sample size of 858 patients.

### Statistical analysis

All data will be collected in a database (Castor) and will be exported to IBM SPSS Statistics (IBM, Armonk, NY, USA) for data analysis. The data analyses will be performed according to the intention to treat principle. However, considering that second-eye surgery can be delayed in ISBCS patients with complications during first-eye surgery, the analyses will be repeated based on a per protocol analysis. As recommended for non-inferiority trials in literature, both analyses will be performed and non-inferiority will be established if both analyses produce the same conclusion [42]. For all analyses, stratification variables (center and axial length) are accounted for by including them in the analysis model and significance levels will be set at 0.05.

#### Baseline characteristics

Baseline characteristics will be presented either as means with standard deviation and 95% confidence intervals, as median with interquartile range, or as frequencies (with percentages), as appropriate.

#### Primary outcome

With regard to the primary endpoint of refractive outcome, the difference in the proportion of second eyes with a refractive outcome ≤1.0 D of target refraction will be analysed using a logistic regression analysis with correction for stratification variables (center and axial length), where a logistic mixed model with center as random factor to account for the hierarchical nature of the data (patients are nested in centers) will be used as sensitivity analysis. To evaluate non-inferiority, a one-sided test procedure will be used. Non-inferiority will be established at the α = 0.05 significance level if the lower limit of a (1-2α)× 100% confidence interval (=90% CI) for the difference (ISBCS - DSBCS) is above the non-inferiority margin (δ) of − 5% [42]. Possible scenarios of observed treatment differences between ISBCS and DSBCS will be evaluated as reported by the CONSORT Statement 2010 [43].

#### Secondary outcomes

For secondary outcomes, data analyses will be performed as follows: presence of non-inferiority of ISBCS compared to DSBCS regarding the difference in the proportion of second eyes with a refractive outcome within ±0.5 D of target refraction will be evaluated similar to the method described under primary outcome. For visual acuity, presence of non-inferiority of ISBCS will be evaluated by comparing the proportion of patients with a corrected distance visual acuity (CDVA) 4 weeks after surgery of the second eye of ≤0.10 LogMAR between groups. The computed sample size of 858 patients for the primary outcome translates to an allowed non-inferiority margin of 10% to establish non-inferiority at the 0.05 alpha level regarding this secondary outcome measure. Corrected and uncorrected distance visual acuity in second eyes, patient-reported outcomes, and utilities will be analysed using a linear mixed model to test for overall differences between groups over time. The difference in the incidence of intraoperative and postoperative complications will be analysed using mixed-effects logistic regression to account for the fact that both eyes of each patient are included in the analysis and nesting of patients within centers.

#### Cost-effectiveness analysis

A trial-based economic evaluation will be performed over a three-month time horizon (first-eye surgery until the final questionnaires at 3 months after first-eye surgery). A societal and healthcare perspective will be used and ICERs will be calculated for the outcome measures described earlier in this protocol. Uncertainty in the point estimates of the ICERs will be assessed using bootstrap analyses with 1000 replications. Based on these bootstrap analyses, cost-effectiveness acceptability curves will be constructed, to show the probability that the intervention is cost-effective for a range of threshold values of the ICER. Additional sensitivity analyses will be performed to investigate the impact of varying input parameters used in the cost-effectiveness analysis.

In addition to the trial-based economic evaluation, the lifetime economic impact of ISBCS compared to DSBCS will be investigated by means of a decision analytical model, in order to take rare events (e.g. bilateral complications) into account. The model-based economic evaluation will address the cost per QALY (societal perspective) and cost per patient with postoperative refraction within 1.0 D from target refraction (health care perspective). Input data for the model will be based on the data from the current study and from a literature review. We will express the model’s robustness and uncertainty by means of sensitivity analyses, confidence intervals, and by creating cost-effectiveness acceptability curves. Annual discount rates of 4 and 1.5% for costs and QALYs, respectively, will be used to determine the present value of costs and QALYs accrued over a lifetime.

In accordance with the Dutch guidelines for economic evaluations, the EQ-5D-5L will be used to determine QALYs using published Dutch tariffs [39]. In addition, the HUI-3 will be used because it is the only generic preference-based HRQL questionnaire that includes questions about vision [40].

For the cost analysis, all relevant costs incurred during the follow-up period from a healthcare and societal perspective will be included. Hospital-based resource use will be identified through patients records. Other resource use (e.g., use of homecare, costs of spectacles, productivity losses) will be measured through the standardized cost questionnaire at baseline and at 3 months after first-eye surgery. Resource use will be valued in accordance with the Dutch guidelines for costs analyses [44]. All costs will be adjusted for inflation to a common price level using the Consumer Price Index.

#### Budget impact analysis

A budget impact analysis (BIA) will be performed to evaluate the impact of implementation of ISBCS on the Dutch healthcare budget. The BIA will be performed in accordance with the Dutch guidelines for economic evaluations and the ISPOR guidelines [45, 46]. The analysis will make use of the budget holder perspective. Additional perspectives include healthcare providers and insurers. A time horizon of 3 years will be used to account for gradual implementation of ISBCS. The BIA will be performed using a simple Microsoft Excel decision analytic model. It will be assumed that the difference in costs will be related completely to substitution of DSBCS by ISBCS. Different scenarios will be compared to investigate various levels of implementation (i.e., 25, 50, 75, and 100% of eligible patients). Sensitivity analyses will be performed to test the robustness of the analysis.

### Data management and monitoring

Personal data will be handled confidentially, according to Good Clinical Practice guidelines (GCP). Data will be collected in an online case report form (CRF), available in the certified electronic data capture tool ‘Castor’ by a member of the study team of each study center. All data will be stored and analysed using only anonymous randomization numbers. Personal data connecting a patient to the anonymous number will be archived by the principal investigator of each study center for a period of 15 years. This study will be monitored by the Clinical Trial Center Maastricht (CTCM) in order to protect patient rights and accuracy of reported trial data. The CTCM is an academic research organisation, familiar with monitoring procedures.

## Discussion

Worldwide, an increasing number of cataract surgeries is performed over the years and healthcare expenditures are rising. Therefore, small efficiency gains in cataract care delivery, such as those potentially gained in ISBCS, may lead to substantial cost savings on a macro level. ISBCS is increasingly practiced on a routine basis in a number of countries around the world, including Finland, certain regions in Spain, and Canada [47]. Potential benefits of ISBCS include less time between surgeries, fewer hospital visits, a faster total recovery period due to simultaneous postoperative care (eye drops) in both eyes and less use of homecare. As a consequence of the recent COVID-19 crisis we now face a scenario where the number of hospital visits, if feasible, should be decreased to diminish the risk for contamination in the hospital. ISBCS could be an instrument to significantly decrease residence time of patients in the clinic for surgery as well as for postoperative controls. However, opponents of ISBCS argue that the potential risks of severe bilateral complications and unexpected refractive outcomes supersede any economic arguments [48]. The main concerns about ISBCS include potential risks of bilateral complications of cataract surgery, most importantly the very rare but severe risk of endophthalmitis and the risk of refractive surprise (a significant deviation from the predicted refraction).

Further investigation is required to evaluate how ISBCS compares to DSBCS with respect to cataract surgery outcomes, in particular with regard to refractive outcomes. In addition, there is a lack of well-designed cost-effectiveness studies using standard methods. The BICAT-NL study will provide more insight in refractive and cost-effectiveness outcomes for ISBCS compared to DSBCS.

### Study status

Currently, recruitment of patients is on-going. Recruitment has started on the 4th of September 2018 and is expected to be completed by August 2020. The latest version of the study protocol is version 7.0 (date: 02-08-2019).

## Data Availability

This manuscript does not contain any data or results. Therefore, data sharing is not applicable.

## Abbreviations

BIA: Budget impact analysis
CDVA: Corrected Distance Visual Acuity
CRF: Case Report Form
CTCM: Clinical Trial Center Maastricht
DSBCS: Delayed Sequential Bilateral Cataract Surgery
ETDRS: Early Treatment Diabetic Retinopathy Study
GCP: Good Clinical Practice
HRQL: Health-related Quality of Life
HUI-3: Health Utility Index Mark 3
ICER(s): Incremental cost-effectiveness ratio(s)
ISBCS: Immediately Sequential Bilateral Cataract Surgery
LogMAR: Logarithm of the Minimum Angle of Resolution
NEI VFQ-25: National Eye Institute Visual Function Questionnaire-25
PROM(s): Patient Reported Outcome Measure(s)
QALY(s): Quality Adjusted Life Year(s)
RCT: Randomised Controlled Trial
